# Placebo response in trials of drug treatments for cancer-related fatigue: a systematic review, meta-analysis and meta-regression

**DOI:** 10.1136/bmjspcare-2019-002163

**Published:** 2020-02-11

**Authors:** Rocio Roji, Patrick Stone, Federico Ricciardi, Bridget Candy

**Affiliations:** 1 Marie Curie Palliative Care Research Department, University College London, London, UK; 2 Department of Statistical Science, University College London, London, UK

**Keywords:** cancer, methodological research, fatigue

## Abstract

**Background:**

Cancer-related fatigue (CRF) is one of the most distressing symptoms experienced by patients. There is no gold standard treatment, although multiple drugs have been tested with little evidence of efficacy. Randomised controlled trials (RCTs) of these drugs have commented on the existence or size of the placebo response (PR). The objective of this systematic review was to establish the magnitude of the PR in RCTs of drugs to relieve CRF and to identify contributing factors.

**Method:**

RCTs were included in which the objective was to treat CRF. A meta-analysis was conducted using the standardised mean change (SMC) between baseline and final measurement in the placebo group. To explore factors that may be associated with the PR (eg, population or drug), a meta-regression was undertaken. Risk of bias was assessed using the revised Cochrane tool.

**Results:**

From 3916 citations, 30 relevant RCTs were identified. All had limitations that increased their risk of bias. The pooled SMC in reduction in fatigue status in placebo groups was −0.23 (95% confidence intervals −0.42 to −0.04). None of the variables analysed in the meta-regression were statistically significant related to PR.

**Conclusion:**

There is some evidence, based on trials with small samples, that the PR in trials testing drugs for CRF is non-trivial in size and statistically significant. We recommend that researchers planning drug studies in CRF should consider implementing alternative trial designs to better account for PR and decrease impact on the study results.

## Background

The placebo response describes the phenomenon whereby patients’ symptoms may improve while receiving an inactive substance. Placebo response is frequently reported in randomised controlled trials (RCT) that assess the effectiveness of a drug against a placebo. The rate of placebo response in RCTs in various mental or physical illnesses has often been found to be around 30%–40%.^1–3^ Although it is important to note some reviews suggest this proportion is increasing. When no evidence-based standard of care exists, the potential existence and frequency of placebo response is a major reason for incorporating in clinical trials a placebo control arm and double-blinding.^4^ The aim in doing so is to allow quantification of the extent to which effects may be attributed specifically to the action of the drug. However, when the placebo response is high, this can challenge the interpretation of the treatment effects.^5^

The placebo response is a complex phenomenon. The mechanisms for it are unclear, although various explanations have been proposed. These include the Hawthorne effect, patients’ expectations about perceived treatment assignment, behavioural conditioning, therapeutic relationship with investigator, regression to the mean and natural fluctuations of the disease (see online supplementary materials).^6^ However, since these effects can also be found in the drug arms of clinical trials, some have questioned the rationale of RCTs.^7^ This argument though is not straightforward as the placebo response may differ between arms of a trial and there are statistical models that seek to overcome this issue.^8^ There are also trial designs that attempt to manage the placebo response.^8–10^ Evidence regarding the benefit of these strategies is not always clear.^10^

10.1136/bmjspcare-2019-002163.supp1 Supplementary data

Cancer-related fatigue (CRF) is one of the most distressing symptoms experienced by patients with cancer and can occur at any stage of the disease and among disease-free cancer survivors.^11^ In some cases, it is possible to find causes for the fatigue that might improve with a specific directed treatment, for example, hypothyroidism, anaemia or depression. However, in most cases (and particularly in advanced disease), fatigue has a multifactorial aetiology. In these circumstances, it is usual to turn to a primarily symptomatic treatment, with multidimensional and interdisciplinary management being preferable.^12^ Although several drugs have been tested to alleviate CRF, there is no approved standard reference treatment. Moreover, from a methodological point of view, it is currently difficult to make a head-to-head comparative analysis of trials because of the diversity of scales used to measure CRF and the small sample sizes of many trials. There are though multiple randomised placebo-controlled double-blind trials of drug treatments for CRF. In these trials including open-label studies, authors frequently refer to the placebo response as being ‘high’. 13 14 Although elsewhere, in the related field of chronic fatigue syndrome, a meta-analysis of 29 RCTs found that the placebo response in trials of drug and behavioural treatments was lower than had been conventionally asserted.^1^ A recent review of placebo response across both drug and non-drug treatments for CRF found a placebo response of 29%.^15^ However, this review was limited in its scope and did not include all of the relevant trials. No previous systematic review seeking evidence from multiple sources and using a robust search strategy has sought to establish more precisely, through meta-analysis, the size of the placebo response in CRF nor, through meta-regression, to identify which factors may influence it. Greater understanding about how fatigue symptoms respond to placebo may provide insights about the nature of fatigue, which in turn may lead to more effective treatments and better care for patients.

## Objectives

To undertake in a systematic review: (1) a meta-analysis to establish the size of the placebo response in drug trials for CRF and (2) a meta-regression to explore factors contributing to the placebo effect.

## Methods

The protocol for this review is registered at PROSPERO (http://www.crd.york.ac.uk/PROSPERO/display_record.php?ID=CRD42018115125).

### Selection criteria

#### Types of studies

Placebo-controlled double-blind RCTs were included. Mixed design interventions, and N-of-1 trials were excluded.

#### Participants

Studies including adults aged 18 years and over who had a clinical diagnosis of cancer and were at any stage of cancer treatment were included.

#### Interventions

RCTs were included that investigated a drug intervention to improve CRF as a prior aim, and where fatigue was assessed by a validated and patient self-reported tool. CRF was defined as fatigue in patients with cancer at any stage, including those on treatment and in disease-free cancer survivors. To be included in the review, RCTs needed to compare drug therapy with a placebo. Standard care or non-pharmacological intervention comparisons were not included. As recommended by recent reviews, due to safety concerns, erythropoietin and darbepoetin should no longer be used for CRF symptomatic treatment.^16^ Therefore, and because they do not represent symptomatic treatment of fatigue but rather treatment of anaemia, we excluded RCTs testing these drugs.

### Primary outcome

We assessed placebo response using standardised mean change (SMC) in fatigue from baseline to study endpoint, defined as the mean change divided by the standardised deviation (SD) in the placebo group. SMC expresses the size of the intervention effect in each study relative to the variability observed in that study. Pretreatment SD is not affected by the nature of treatment and constitutes a straightforward metric to use.^17^ The calculation of SMC does not correct for differences in the direction of the scale. For some scales, an increased scale score indicates a reduction in fatigue severity, while for other scales, the opposite is true. In the latter cases, we therefore multiplied the mean values by −1 to ensure than all the scales pointed in the same direction.

### Search methods for identification of studies

The search strategy included medical subject headings and text terms to describe population, intervention, study design and care setting (see online supplementary materials for further details). The sensitivity of the search strategy was tested by identifying key references from other reviews in the field of CRF. There were no date restrictions. Studies in any language were eligible.

Seven databases were searched to 15 November 2018: MEDLINE, Embase, Cochrane Central Register of Controlled Trials, PsychInfo, CINAHL and WHO clinical trials registry. online supplementary materials provide search strategy details. Citations from articles obtained were screened independently by two authors (RR and BC) against the inclusion criteria. Where it was not possible to exclude a study, we retrieved full text. At full text, we recorded reasons for exclusion of any studies subsequently found not to fulfil our inclusion criteria.

### Data collection and analysis

#### Data extraction

For all studies included, data were extracted on key characteristics by one reviewer (RR) and a second (BC) checked entries. If the studies presented results for intention to treat or per protocol, we used this presentation in order to include all participants randomised, thereby reducing bias and using the data that were most similar to the real-world clinical situation. We tried to contact authors when necessary to request any essential missing data.

#### Assessment of risk of bias in included studies

Two review authors (BC and RR) independently assessed risk of bias for each trial using the Revised Cochrane risk-of-bias tool for randomised trials (RoB2).^18^ We resolved any disagreements by discussion. Bias was assessed per five domains as either high risk, low risk or where there were some concerns. The domains were:

1. Randomisation process.
2. Deviations from intended interventions.
3. Missing outcome data.
4. Measurement of the outcome.
5. Selection of the reported result.

The approach to mapping risk-of-bias judgements within domains to an overall judgement was done following RoB2 tool. Judging a result to be at a particular level of risk of bias for an individual domain implies that the result has an overall risk of bias at least this severe. Therefore, a judgement of ‘high’ risk of bias within any domain has similar implications for the overall result, irrespective of which domain was assessed. Where the risked is judged as ‘some concerns’ in multiple domains lthe review authors givie an overall judgement of ‘High’ risk of bias for that outcome or group of outcomes.

#### Meta-analysis and meta-regression

Due to the likely high degree of study heterogeneity, meta-analysis was conducted by estimating both fixed and random effects models for SMC. Heterogeneity across studies was quantified using the I^2^ statistic and the between study variance τ2, calculated using the restricted maximum likelihood estimator. The Q-statistic, which follows a χ^2^ distribution with K-1 df (with K being the number of studies considered), was used to test whether heterogeneity was statistically significant. Based on Cochrane guidelines, we provided an overall judgement on heterogeneity based on the I^2^ thresholds^18^:

- 0%–40%: low or might not be important.
- 40%–60%: may represent moderate heterogeneity.
- 60%–90%: may represent substantial heterogeneity.
- 90%–100%: may represent considerable heterogeneity.

If heterogeneity was not found to be important, a fixed effect model was to be reported; if heterogeneity was found to be important, then a random effects model was to be reported.

Publication bias was assessed using a funnel plot.

Meta-regression analyses were conducted to investigate the role of various factors that may affect placebo response and in doing would seek to provide reasons for any statistical heterogeneity found across the trials. Following group discussion prior to analysis, we selected certain participants features (age, gender and cancer stage) and study features of duration, risk of bias, number of sites, number of participants in the placebo arm, study type (eg, cross-over or parallel), year, fatigue measurement tool and drug group (eg, psychostimulants or herbs). Placebo dosing regimen (fixed dose/flexible dose) was also selected because it has been found to be associated with placebo response in other conditions such a depression.^19^ In addition, sample size was selected because it has been reported as a factor influencing placebo response in RCTs, with small studies overestimating the effect sizes in meta-analyses.^20^ We also considered tools used to measure fatigue. We included the fatigue assessment tool as a variable in our meta-regression because we had found that it was most commonly used in the included studies. Type of trial was not originally included as one of the proposed variables in our meta-regression. However, during the review, we noted some studies suggesting that combining shams and sequences can prejudice the conclusions in cross-over designs.^21^ This bias seems to increase the risk of rejecting potentially valid treatments. Moreover, other meta-regression studies have included similar variables such as ‘number of arms’ or ‘washout period’. For these reasons, we decided to include type of study as one of the variables in our meta-regression. All statistical procedures were performed using the R statistical package (version 3.4.3).

## Results

### Results of the search

**Figure 1:**
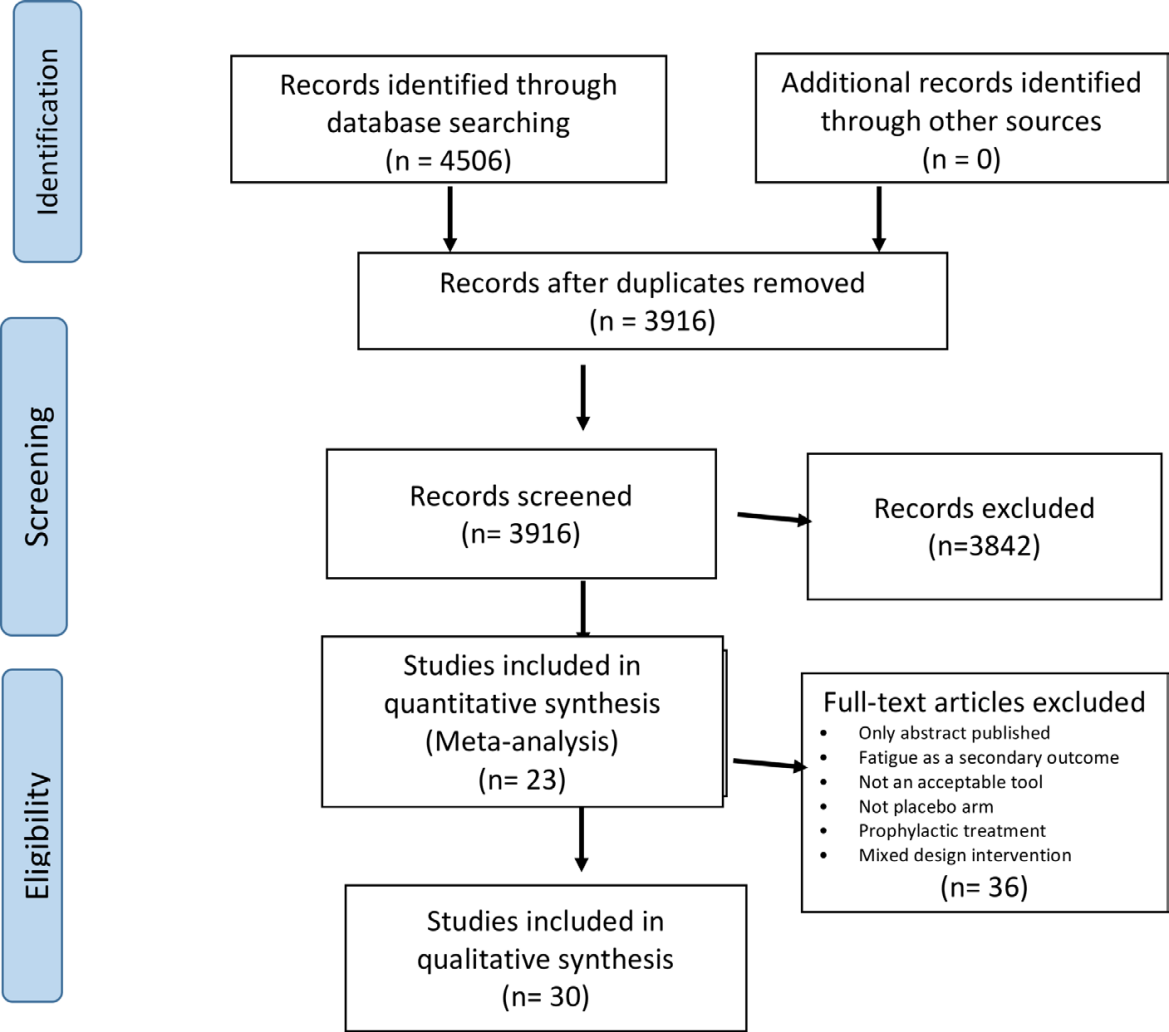
PRISMA flow diagram. PRISMA, Preferred Reporting Items for Systematic Reviews and Meta-Analyses.

The search identified 3916 unique references. At screening, 74 potentially relevant citations were identified. All authors discussed full text of these citations and agreed that 37 trials potentially fulfilled inclusion criteria. The main reasons for rejecting articles were: fatigue was a secondary outcome; no self-reported fatigue measurement; no placebo arm; the intervention was for the prevention (rather than the treatment) of fatigue; or it was a mixed design intervention (with no clear placebo-only arm or design N of 1). In further exploration at data extraction, seven more studies were rejected for one or more of the reasons listed above. The final number of trials included for qualitative analysis was 30 22–51 (figure 1). In 11 trials, some of the data needed for meta-analysis were not published in the trial paper; therefore, we sought contact with authors. We retrieved the necessary information from four of the 11 studies, making the number of trials available for quantitative analysis 23^22–29 31–34 39 41–47 49–51^ (online supplementary material for details on rejected articles).

### Study characteristics

Two groups of drug therapies were most commonly studied. Psychostimulants including modafinil, armodafinil, dexamphetamine, dexmethylphenidate and methylphenidate accounted for 43% of the drugs studied. Herbs such as guarana, ginseng and PG2 (infusible botanically derived drug) made up 26%. Other drugs formed a miscellaneous group consisting of antidepressants (paroxetine and bupropion), hormones (thyrotropin-releasing hormone (TRH) and testosterone), steroids, carnitine, melatonin and donepezil (table 1).

**Table 1:** Characteristics of studies included

Study	Intervention	Study design	Number of sites	Age in years, mean (SD) placebo arm	% males placebo arm	Tool to measure fatigue	Weeks	Placebo dosage	Route	Cancer stage	N
Ashrafi *et al* 22	Bupropion	Two arms	ns	55.2 (17.6)	50	FACIT-F	4	Once- a day	Oral	Palliative	20
Auret *et al* 23	Dexamphetamine	Two arms	1	67.8 (12.5)	80	BFI	1	Twice a day	Oral	Palliative	18
Barton *et al* 24	Ginseng	Four arms	ns	62 (13)	35	BFI	8	Twice a day	Oral	Curative	39
Barton *et al* 25	Ginseng	Two arms	40	55.9 (11.8)	25	MFSI-SF	4	Twice a day	Oral	Survivors	153
Berenson *et al* 26	Armodafinil	Cross-over		67 (2.3)	60	BFI	4	Once a day	Oral	Curative	23
Boele *et al* 27	Modafinil	Cross-over	3	ns	ns	CSI	6	Twice a day	Oral	Curative	14
Bruera *et al* 28	Donepezil	Two arms	2	56 (12.4)	35.2	FACIT-F	1	Once a day	Oral	Palliative	56
Bruera *et al* 29	Methylphenidate	Two arms	2	ns	41.5	FACIT-F	1	prn	Oral	Palliative	53
Chen *et al* 30	PG2	Two arms	1	56.9 (ns)	37	BFI	4	Less than once a day	Intravenous	Palliative	30
Cruciani *et al* 31	Carnitine	Two arms	2	70.3 (12.9)	33	FACT-An	2	Twice a day	Oral	Palliative	12
Cruciani *et al* 32	Carnitine	Two arms	24	62 (12.2)	42.3	BFI	4	Twice a day	Oral	Curative	138
de Oliveira *et al* 33	Guarana	Cross-over†	2	ns	0	FACIT-F	3	Twice a day	Oral	ns	60
Del Fabbro *et al* 34	Testosterone	Two arms	2	63 (6)	100	FACIT-F	4	Less than once a day	IM	Palliative	16
del Giglio *et al* 35	Guarana	Two arms	52.17	15.17	31.2	BFI	3	Twice a day	Oral	Any stage	40
Eguchi *et al* 36	Corticosteroids	Two arms	22	ns	62.5	VAS	1	Twice a day	Oral	Palliative	16
Escalante *et al* 37	Methylphenidate	Two arms	1	ns	0	BFI	4	Once a day	Oral	Curative	33
Hovey *et al* 38	Modafinil	Two arms	25	68 (10.7)	78.6	MDASI	2	Twice a day	Oral	Curative	24
Jean-Pierre *et al* 39	Modafinil	Two arms	2	60 (ns)	34.1	BFI item 3	8	Progressive	Oral	Curative	316
Kamath *et al* 40	TRH	Cross-over	2	58 (9.4)	12.5	VAS-E		Less than once a day	Intravenous	Curative	8
Lee *et al* 41	Armodafinil	Two arms	ns	ns	53.8	FACIT-F	6	Once a day	Oral	Palliative	29
Lower *et al* 42	Dexmethylphenidate	Two arms	24	53.2 (8.4)	6.5	FACIT-F	8	Twice a day	Oral	Curative	77
Lund Rasmussen *et al* 43	Melatonin	Cross-over†	1	60/64 (ns)		MFI-20	2	Once a day	Oral	Palliative	50
Moraska *et al* 44	Methylphenidate	Two arms		60.6 (13.8)	43	BFI	2	Progressive	Oral	Curative	63
Morrow *et al* 45	Paroxetine	Two arms	18	56.3 (12.3)	28	MAF question 1	8	Once a day	Oral	Curative	235
Richard *et al* 46	Methylphenidate	Two arms	1	ns	100	FACIT-F	10	Progressive	Oral	Curative	12
Roth *et al* 47	Methylphenidate	Two arms		71 (10)	100	BFI	6	progressive	Oral	Palliative	13
Sette *et al* 48	Guarana	Two arms	1	55.7 (ns)	0	BFI	5	Twice a day	Oral	Curative	5
Sette *et al* 48	Guarana	Two arms	1	52 (ns)	0	BFI	3	Twice a day	Oral	Curative	25
Spathis *et al* 49	Modafinil	Two arms	24	69.1 (9.4)	50.5	FACIT-F	4	Progressive	Oral	Palliative	85
Yennurajalingam *et al* 50	Dexamethasone	Two arms	3	ns	59.9	FACIT-F	2	Twice a day	Oral	Palliative	41
Yennurajalingam *et al* 51	Ginseng	Two arms	1	n*s*	62.5	FACIT-F	4	Twice a day	Oral	Palliative	56

*Cross-over design, but endpoint before cross-over, so studied as two arms.

†Cross-over with washout period, all placebo data were used.

BFI, Brief Fatigue Inventory; CSI, Checklist Individual Strength; FACIT-F, Functional Assessment of Chronic Illness Therapy – Fatigue; FACT-An, Assessment of Cancer Therapy – Anemia; IM, intramuscular; MAF, Multidimensional Assessment of Fatigue; MDASI, MD Anderson Symptom Inventory; MFI, Multidimensional Fatigue Inventory; MFSI-SF, Multidimensional Fatigue Symptom Inventory – Short Form; ns, not stated; TRH, thyrotropin-releasing hormone; VAS, Visual Analogue Scale; VAS-E, Visual Analog Scale-Energy.

The fatigue scales used as primary outcome measures most commonly were the Functional Assessment of Chronic Illness Therapy-Fatigue in almost half of the studies and the Brief Fatigue Inventory in around 25%. Other tools used were the Multidimensional Fatigue Symptom Inventory – Short Form, Checklist Individual Strength, Assessment of Cancer Therapy – Anaemia, Multidimensional Fatigue Inventory and Multidimensional Assessment of Fatigue. The most common type of trial (84%) was a parallel two-arm study with a median duration of 4 weeks’ treatment. The proportion of women was slightly higher than the proportion of men, and the mean age of the participants was 60 years. Forty-six per cent of studies involved solely participants at a curative stage of treatment and another 46% at a palliative stage. In one of the other studies participants were survivors’ and in another participants had cancer at any stage. One paper did not report cancer stage (table 1).

### Risk of bias assessment in those included in meta-analysis

Nine of the studies had overall a low risk of bias, for eight some concerns relating to risk were found and in six studies the risk of bias was high (table 2).

**Table 2:** Risk-of-bias assessment in those included in meta-analysis/regression

	Randomisation	Deviation from intended interventions	Missing outcome data	Measurement outcome	Selection of reported result	Overall risk of bias (RoB2)
Ashrafi *et al* 22						
Auret *et al* 23						
Barton *et al* 24						
Barton *et al* 25						
Berenson *et al* 26						
Boele *et al* 27						
Bruera *et al* 28						
Bruera *et al* 29						
Cruciani *et al* 31						
Cruciani *et al* 32						
de Oliveira *et al* 33						
Del Fabbro *et al* 34						
Jean-Pierre *et al* 39						
Lee *et al* 41						
Lower *et al* 42						
Lund Rasmussen *et al* 43						
Moraska *et al* 44						
Morrow *et al* 45						
Richard *et al* 46						
Roth *et al* 47						
Spathis *et al* 49						
Yennurajalingham *et al* 30						
Yennurajalingham *et al* 51						

Yellow=some concerns, red=high risk of bias, green=low risk of bias.

RoB2, revised Cochrane risk-of-bias tool for randomised trials.

### Response in placebo arm: meta-analysis and meta-regression

**Figure 2:**
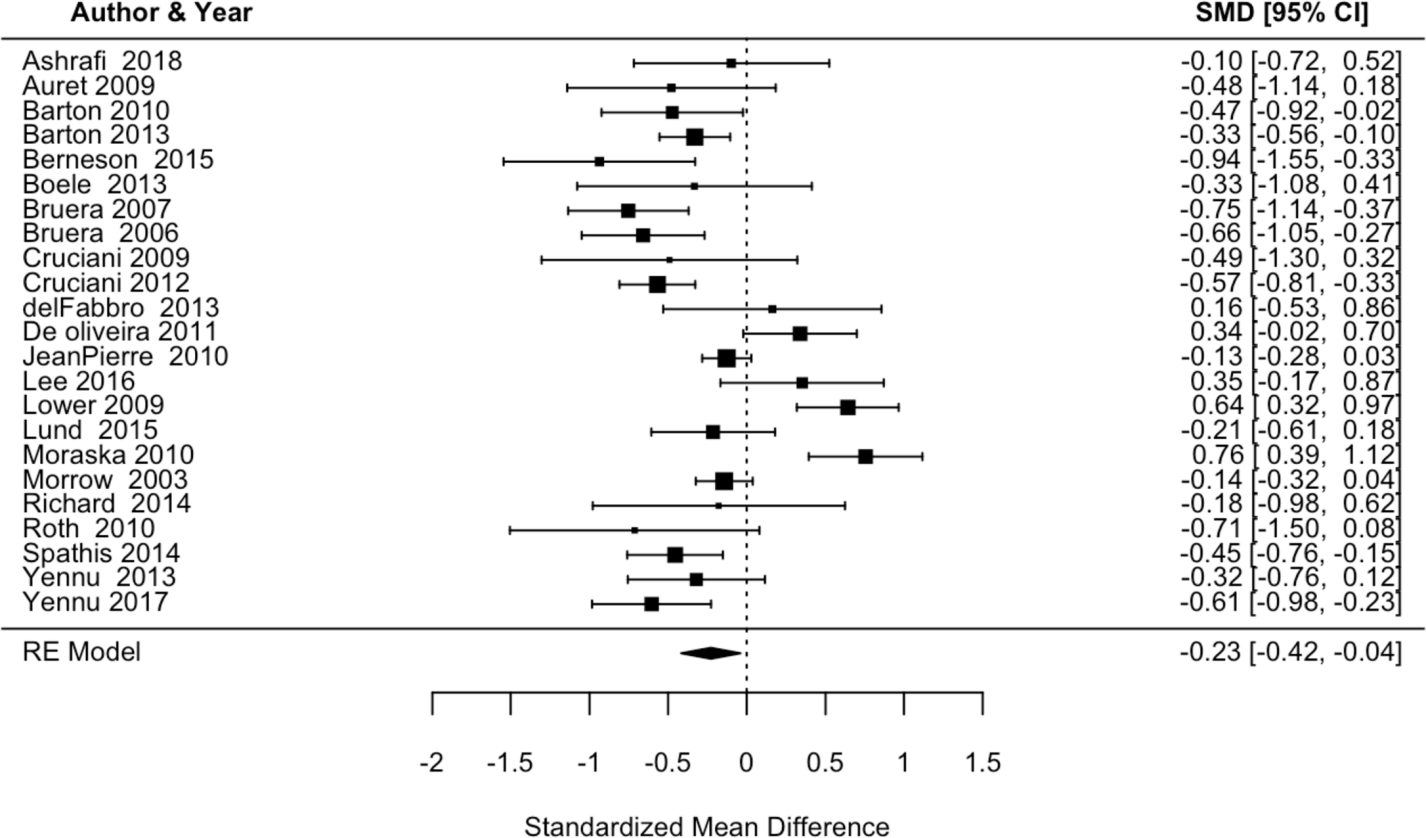
Forest plot of random effect model standardised mean change fatigue in placebo arm.

**Figure 3:**
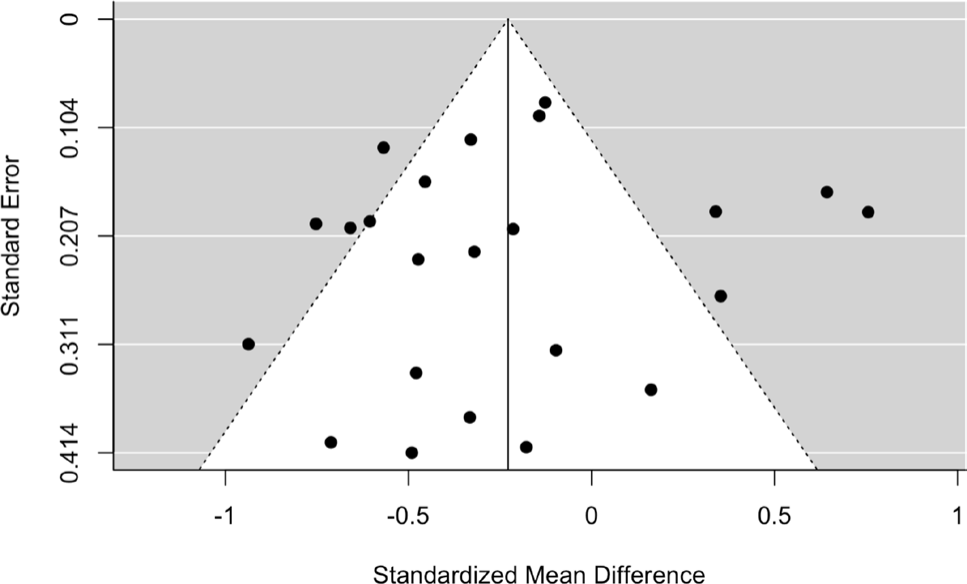
Funnel plot.

Results from the meta-analysis are detailed in figure 2. From 23 trials of 1582 participants in total, the pooled estimate SMC reduction in fatigue was −0.23 (95% CI −0.42 to −0.04). The use of a random effect model is reasonable due to substantial heterogeneity across the studies, as highlighted by I^2^=78.4% (95% CI 70.4% to 86.2%) and significant Q-statistic (109, p<0.0001). The symmetric-shaped funnel plot suggests little risk of publication bias, although some midsized studies have a relatively large SMC, falling outside the funnel area (figure 3).

We used meta-regression to determine which study characteristics may, in part, explain such dispersion. Due to the limited number of studies considered, it was not possible to explore multiple characteristics and their interactions. Instead, we examined these factors independently, fitting separate regression models, one for each covariate of interest (table 3). Not all the studies reported all the mediators of interest, so if a study was missing a covariate, it was omitted from that regression model. None of the covariates were found to be statistically significant factors.

**Table 3:** Meta-regression results

Mediators		Coefficient	2.50%	97.50%	P value
Study year		−0.002	−0.05	0.05	0.94
Mean age		−0.039	−0.08	0.00	0.07
% males		0.004	−0.00	0.01	0.47
Study length in weeks		0.04	−0.03	0.11	0.29
Risk of bias (low is the reference level)	Some concerns	−0.094	−0.55	0.36	0.68
	High	0.038	−0.45	0.53	0.87
Stage (curative is the reference level)	Palliative	−0.307	−0.71	0.10	0.14
Number of sites		0.003	−0.01	0.01	0.67
Type of study (parallel arms trial is the reference level)	X-over	−0.009	−0.52	0.51	0.97
Subjects in the placebo arm (categorical variable, 0–50 is the reference level)	51–199	0.117	−0.	0.53	0.58
	200+	0.165	−0.49	0.82	0.62
Subjects in the placebo arm (continuous variable)		0	−0.002	0.003	0.74
Tool item (single is the reference level)	Multidimensional	−0.102	−0.73	0.53	0.75
Placebo dosage regimen (fixed dose is the reference level)	Flexible dose	−0.001	−0.44	0.43	0.99
Type of drug (other is the reference level)	Psychostimulants	0.157	−0.22	0.53	0.41

## Discussion

This review set out to establish what is known from the current evidence on the placebo response in trials testing drugs for CRF. We found that placebos had a statistically significant and a non-trivial impact on reducing fatigue (SMC −0.23, p=0.02). Meta-regression did not identify explanatory factors. Both results, however, should be treated with caution. The studies were of limited number and quality. There was, across trials, high statistical heterogeneity (I^2^=78.4%).

In a Cochrane review for pain and nausea, similar effect sizes were found in meta-analyses.^17^ Although other reviews have found larger effect size for placebos, a meta-analysis of placebo response in antipsychotic trials found a magnitude of response of SMC −0.33.^5^ Many factors have been proposed as placebo response influencers (as shown in online supplementary materials). Meta-regression in depression and neuropathic pain has also been used to identify specific predictors of the placebo response, including the severity of symptoms and dosing schedules. A review of two specific CRF drug trials identified that worse baseline physical well-being was associated with placebo response.^52^ While no factors related to placebo response were identified in our current study, this may be a consequence of the small sample sizes in the included trials. It may also be a limitation of the variables extracted from the included studies or other factors related to fatigue not included in this review.

### Narrative review of RCTs referring to placebo response

Nine of the 23 included studies referred to the placebo response to explain their findings. We examined these papers in detail to identify any distinctive features of the trial design. The most common characteristic was that four of the studies were interventions using psychostimulants.^26 27 47 49^ Our meta-regression, in contrast, did not find psychostimulants as a factor in predicting placebo effect. However, our regression only involved a limited number of studies. When new psychostimulant trials are available, this could be explored further.

### Strengths and limitations

To our knowledge, this is the first systematic review of the placebo response in drug interventions for CRF. International standards were used in review processes, including critique and analysis. Contact was sought with authors for missing data.

One of the limitations is that the principal outcome of the meta-analysis was not the main outcome of any of the included studies (which were designed to measure drug response not placebo response), and consequently the data needed for our analysis were not available in all trials. The number of studies available for meta-analysis and meta-regression was not large (n=23); moreover, the sample sizes of the studies included was small, with only two studies including more than 200 patients in the placebo arm. Of the seven studies that fulfilled our inclusion criteria but did have data available for our analysis, four referred to a placebo effect. Therefore, the effect size from the meta-analysis may be an underestimate. These limitations mean that our results are exploratory as they are not based on the strongest level of evidence.

### Implications for future research

#### Does it matter if the placebo response is high in RCTs?

We found that a number of authors have sought to explain the negative findings of their trials by referencing a high placebo response rate. Although it is not clear that decreasing the placebo response rate would make it any easier to demonstrate the superiority of the intervention arm over the placebo treatment,^53^ some authors have suggested ways in which the placebo response could be reduced. One of the most common suggested strategies is to include a run-in phase, in which placebo responders are withdrawn before random assignment to treatment condition. However, in depression, this approach has been found not to demonstrate any difference in placebo effect sizes compared with trials without a placebo run-in phase.^53^ Design measures that would reduce the placebo response in the control arm would also reduce the proportion of participants in the intervention arm who reported ‘non-specific’ (or placebo) responses, while measures that may be used to reduce the placebo response may also decrease the response to the drug. Moreover, study designs that lower the placebo response may make the results less applicable to wider populations and real-world clinical settings.^9^ The science of placebo response has grown substantially in the last decades including multiple strategies to manage the response such as no-treatment-control arm trials or enrichment designs. In regards to the placebo response in the active and control arm, it has been suggested though that this impact may differ per arm, and statistical approaches have been developed to accommodate this.^8^

#### Design of future trials

When considering phase II trials or feasibility studies for new drugs in the treatment of CRF, researchers should take into account our results. Researchers planning drug studies in CRF should consider implementing alternative designs to better account for placebo effects and decreasing their impact. Alternative or more advanced statistical methods should also be considered to control for the placebo effect when estimating the treatment effect under classical RCTs.^54^ Introducing qualitative measures in clinical trials can provide a better understanding of fatigue and its palliation. Participant-centred factors (expectations, therapeutic relationship and meaning) and/or those related to the context of care could explain some placebo responses. This strategy could include asking participants closed questions, such as which treatment or placebo they think they received after unblinding, as well as open questions in order to identify other components of care that may have improved outcomes. In CRF studies, this may include other aspects of ‘usual care’ provided in such circumstances, such as counselling, which may help participants to adapt to their fatigue, or additional attention that they received by being a trial participant.

The effects of a positive clinical encounter may exist regardless of blinding of investigators. Researchers should therefore try to identify other components of care that improve outcomes after clinical encounters, since the mechanisms underlying the placebo effect are not fully understood. Ultimately, understanding the way in which placebos affect CRF may help us to develop new non-pharmacological interventions to manage this symptom.^4^

Future research should also focus on the phenomenon of placebo response, what it is and which factors favour it, instead of trying to reduce it. This could help to improve the interventions that are offered to patients with CRF, by maximising the placebo response, so that active treatments are able to produce the most beneficial effects.^55^

## Conclusions

There is some evidence, based on trials with small samples, that the placebo response in trials testing drugs for CRF is non-trivial and is statistically significant. No factors related to placebo response were identified in our study.
