# Supplementary material for: Placebo response in trials of drug treatments for cancer-related fatigue: a systematic review, meta-analysis and meta-regression
Source: BMJ Support Palliat Care. 2020 Feb 11;10(4):385–94. doi: 10.1136/bmjspcare-2019-002163 (PMC7691807; doi:10.1136/bmjspcare-2019-002163)
Supplement: Supplementary data [file bmjspcare-2019-002163supp001.pdf]

Supplementary materials

Table 1. Explanations that have been proposed for placebo response
Box 1. Search strategies used
Box 2. Rejected studies with reason per study
Table 2. Descriptive summary of study characteristics
Table 3 Details of categorical and binary variables used in meta-regression

Roji R, *et al. BMJ Support Palliat Care* 2020; 10:385–394. doi: 10.1136/bmjspcare-2019-002163

Table 1. Explanations that have been proposed for placebo response[1,2,3]

		**Examples**
Patients characteristics	Factors related to recruitment	Methods, source, types
	Severity of symptoms	Participants with mild symptoms are more likely to experience spontaneous recovery
	Duration of symptom	Chronic conditions, acute conditions, fluctuations
	Psychological phenomena	Patient expectations, conditional responses
Investigator factors	Experience	Clinical and conducting trials
	Methods to accelerate enrolment	Financial incentives
	Their availability	Attention perceived by patient
	The degree of positivity which a treatment is presented	Psychological
	Rosental effect	If investigators communicate their expectations to participants, this can alter their behaviour to conform to these expectations
Research design and statistical	Duration of the trial	Participants with recent symptoms are more likely to experience spontaneous recovery
	Number of treatment arms	
	Flexible vs fixed dosage	
	Outcome measurement	Reliability, validity, type and extent of assessments
	Hawthorne effect	Clinical trial participation may alter the behaviour and experience of participants
	Unidentified parallel interventions	
	Regression to the mean	Symptoms that fluctuate and are at their most severe at the initiation of the study may improve spontaneously as a part of their natural history
Placebo related	Use of equipment	Infusions pumps
	Appearance	Shape, colour, number of pills

[1]Sanderson, C., Hardy, J., Spruyt, O. & Currow, D. C. Placebo and Nocebo Effects in Randomized Controlled Trials: The Implications for Research and Practice. (2013).

[2]Dworkin, R. H., Katz, J. & Gitlin, M. J. Placebo response in clinical trials of depression and its implications for research on chronic neuropathic pain. *Neurology* **65,** S7-19 (2005).
[3]Miro Jakovljevic, The placebo–nocebo response: Controversies and challenges from clinical and research perspective, *European Neuropsychopharmacology*, **24**, 3, (333), (2014).

Box 1. Search strategies used

Embase

1 fatigue.mp. or cancer fatigue/ or fatigue/
2 neoplasm/co, dt, rt, rh, si, th [Complication, Drug Therapy, Radiotherapy, Rehabilitation, Side Effect, Therapy]
3 malignant neoplasm/co, dm, dt, rt, rh, si, th , [Complication, Disease Management, Drug Therapy, Radiotherapy, Rehabilitation, Side Effect, Therapy]
4 clinical trial/
5 controlled study/
6 random*.mp.
7 placebo/ct, ad, cm, dt [Clinical Trial, Drug Administration, Drug Comparison, Drug Therapy]
8 2 or 3
9 4 or 5 or 6 or 7
10 1 and 8 and 9
11 Limit 10 to human

Medline

1 FATIGUE/ or fatigue.mp.
2 Neoplasms.mp or NEOPLASMS/
3 Cencer.mp. or Neoplasms/
4 Malignan*.mp.
5 Clinical trial/
6 RANDOM ALLOCATION/
7 Placebo.mp.
8 2 or 3 or 4
9 5 or 6 or 7
10 1 and 8 and 9
11 Limit 10 to humans
12 Limit 11 to “all adult (19 plus years)”

PsychInfo

1 exp FATIGUE/
2 exp NEOPLASMS/
3 cancer.mp. or exp Neoplasms/
4 exp Clinical Trials/
5 exp RANDOM SAMPLING/
6 exp Drug Therapy/ or exp Placebo/ or exp Clinical Trials/ or exp Drugs
7 2 or 3
8 4 or 5 or 6
9 1 and 7 and 8
10 Limit 9 to human
11 Limit 10 to adulthood <18+ years>

CINAHL plus

S1 fatigue
S2 fatigue management
S3 neoplasms or oncology or cancer
S4 clinical trials or randomized controlled trials
S5 S1 or S2
S6 S3 and S4 and S5, S7 limits

Box 2. Rejected studies with reason per study

Studies rejected	Reason for rejection
Barton 2011[1]	Secondary outcome using BFI.
Beijer 2010 [2]	Not placebo arm. ATP vs usual care.
Beniwal 2015[3]	Only Abstract, not published, not data enough.
Boele 2013[4]	Abstract of full paper included in review.
Bruera 2013[6]	Mixed design intervention.
Butler 2007[7]	Prophylactic.
Conley 2016[8]	Secondary analysis of paper included in review
Cornish 2015[10]	Capacity and function related to exercise +BFI.
Cruciani 2004[11]	No placebo controlled.
Cruciani 2006[12]	No placebo controlled.
daCostaMiranda 2009[13]	Prophylactic treatment.
DelFabbro 2011[14]	Abstract of full paper included in review.
DelFabbro 2012[16]	Abstract of full paper included in review.
delFabbro 2013[17]	Fatigue as a secondary outcome.
deSouza 2007[18]	Prophylactic.
Dimsdale 2011[19]	Secondary outcome.
Djalali 2013[20]	No abstract available.
Heckler 2016I[21]	Fatigue as a secondary outcome.
Heim 2012[22]	Abstract, and some results posted in German, no published.
Hovey 2012[23]	Abstract of full paper included in review.
Kamath 2012[25]	Abstract of full paper included in review.
Lai 2013[27]	Same study that Chen[28].
Lesser 2012[29]	Prophylactic. And both arms have vit E.
Littlewood 2006D[30]	EPO.
MarFan 2008[31]	Prophylactic.
Mitchell 2015[32]	N-of-1.
Norager2006[33]	EPO.
Page2015[34]	Prophylactic. Fatigue as a secondary outcome.
Palesh2012[35]	No fatigue, secondary analysis of Morrow2003Paroxetine[36].
Paulsen2014[37]	Fatigue as a secondary outcome (ESAS+QoLC30).
Ribeiro2017[38]	Prophylactic treatment.
Richard2014[39]	Abstract of full paper included in review.
Roscoe2005P [41]	Report of paper included in review
Schmidt2012[42]	Abstract not published as an article.
Tanioka2018[43]	Prophylactic treatment.
Yennu2015G[44]	Preliminary report of paper included in review.

Roji R, *et al. BMJ Support Palliat Care* 2020; 10:385–394. doi: 10.1136/bmjspcare-2019-002163

Refeences of excluded studies

1. Barton, D. L. *et al.* The use of Valeriana officinalis (Valerian) in improving sleep in patients who are undergoing treatment for cancer: a phase III randomized, placebo-controlled, double-blind study (NCCTG Trial, N01C5). *J. Support. Oncol.* **9,** 24–31 (2011).
2. Beijer, S. *et al.* Randomized Clinical Trial on the Effects of Adenosine 5•-Triphosphate Infusions on Quality of Life, Functional Status, and Fatigue in Preterminal Cancer Patients. *J. Pain Symptom Manage.* **40,** 520–530 (2010).
3. Beniwal, S., et al. (2015). 'A Phase III randomized, placebo-controlled trial evaluating cancer-related fatigue in patients with advanced cancer treated with dexamethasone.' *Support. care cancer. 23(1 SUPPL. 1) S85.*
4. Boele, F., et al. (2013). ". The effect of modafinil on fatigue, cognitive functioning and mood in primary brain tumor patients: a multi-center RCT.". *Support. care cancer. 21 S148-S149.*
5. Boele, F. W. *et al.* The effect of modafinil on fatigue, cognitive functioning, and mood in primary brain tumor patients: a multicenter randomized controlled trial. *Neuro. Oncol.* **15,** 1420–8 (2013).
6. Bruera, E. *et al.* Methylphenidate and/or a nursing telephone intervention for fatigue in patients with advanced cancer: a randomized, placebo-controlled, phase II trial. *J. Clin. Oncol.* **31,** 2421–2427 (2013).
7. Butler, J. M. *et al.* A phase III, double-blind, placebo-controlled prospective randomized clinical trial of d-threo-methylphenidate HCl in brain tumor patients receiving radiation therapy. *Int. J. Radiat. Oncol. Biol. Phys.* **69,** 1496–501 (2007).
8. Conley, C. C. *et al.* Modafinil Moderates the Relationship Between Cancer-Related Fatigue and Depression in 541 Patients Receiving Chemotherapy. *J. Clin. Psychopharmacol.* **36,** 82–5 (2016).
9. Jean-Pierre, P. *et al.* A phase 3 randomized, placebo-controlled, double-blind, clinical trial of the effect of modafinil on cancer-related fatigue among 631 patients receiving chemotherapy: a University of Rochester Cancer Center Community Clinical Oncology Program Research bas. *Cancer* **116,** 3513–3520 (2010).
10. Cornish, R. S., Bolam, K. A. & Skinner, T. L. Effect of caffeine on exercise capacity and function in prostate cancer survivors. *Med. Sci. Sports Exerc.* **47,** 468–475 (2015).
11. CRUCIANI, R. A. *et al.* L-carnitine supplementation for the treatment of fatigue and depressed mood in cancer patients with carnitine deficiency: a preliminary analysis. *Ann. N. Y. Acad. Sci.* **1033,** 168–176 (2004).
12. Cruciani, R. A. *et al.* Safety, tolerability and symptom outcomes associated with L-carnitine supplementation in patients with cancer, fatigue, and carnitine deficiency: a phase I/II study. *J. Pain Symptom Manage.* **32,** 551–559 (2006).
13. Da Costa Miranda, V. *et al. Effectiveness of Guaraná (Paullinia cupana) for Postradiation Fatigue and Depression: Results of a Pilot Double-Blind Randomized Study*.
14. Del Fabbro, E., et al. 'A randomized placebo-controlled trial of testosterone replacement for fatigue in male hypogonadic patients with advanced cancer.' *J. Clin. Oncol. 29(15 SUPPL. 1).*
15. Del Fabbro, E. *et al.* Testosterone replacement for fatigue in hypogonadal ambulatory males with advanced cancer: a preliminary double-blind placebo-controlled trial. *Support. Care Cancer* **21,** 2599–2607 (2013).
16. Del Fabbro, E., et al. 'Testosterone replacement for fatigue in male hypogonadic patients with advanced cancer: a preliminary double-blind, placebo-controlled trial.' *Support. care cancer. 20 S249-S250.*
17. Del Fabbro, E., Dev, R., Hui, D., Palmer, L. & Bruera, E. Effects of melatonin on appetite and other symptoms in patients with advanced cancer and cachexia: a double-blind placebo-controlled trial. *J. Clin. Oncol.* **31,** 1271–1276 (2013).
18. de Souza Fede, A. B. *et al.* Multivitamins do not improve radiation therapy-related fatigue:

results of a double-blind randomized crossover trial. *Am. J. Clin. Oncol.* **30,** 432–436 (2007).

19. Dimsdale, J. E. *et al.* Effect of eszopiclone on sleep, fatigue, and pain in patients with mucositis associated with hematologic malignancies. *Support. Care Cancer* **19,** 2015–2020 (2011).
20. Djalali, S. [Ginseng in cancer-induced fatigue - more than a placebo]. *Ginseng gegen krebsbedingte Mudigkeit - mehr als ein Placebo.* **102,** 1553–1554 (2013).
21. Heckler, C. E. *et al.* Cognitive behavioral therapy for insomnia, but not armodafinil, improves fatigue in cancer survivors with insomnia: a randomized placebo-controlled trial. *Support. Care Cancer* **24,** 2059–2066 (2016).
22. M.E., H. *et al.* Randomized placebo-controlled double-blind study of modified methylphenidate on cancer-related fatigue (CRF). *J. Cancer Res. Clin. Oncol.* **138,** 13 (2012).
23. Hovey, E. J., et al. (2012). 'Modafinil for fatigue associated with docetaxel-based chemotherapy: a randomized controlled trial.' *J. Clin. Oncol. 30(5 SUPPL. 1) (no pagination).*
24. Hovey, E. *et al.* Phase III, randomized, double-blind, placebo-controlled study of modafinil for fatigue in patients treated with docetaxel-based chemotherapy. *Support. Care Cancer* **22,** 1233–42 (2014).
25. Kamath, J., et al. (2012). 'Thyrotropin-releasing hormone (TRH) as a treatment for cancerrelated fatigue: impact on sleep, quality of life, and immune function.' *Support. care cancer. 20 S45-S46.*
26. Kamath, J., Feinn, R. & Winokur, A. Thyrotropin-releasing hormone as a treatment for cancer-related fatigue: a randomized controlled study. *Support. Care Cancer* **20,** 1745–1753 (2012).
27. Lai, Y., et al. (2013). 'Innovation of pharmacotherapy to cancer-related fatigue.' *Support. care cancer. 21 S89-S90.*
28. Chen, H.-W. *et al.* A novel infusible botanically-derived drug, PG2, for cancer-related fatigue: a phase II double-blind, randomized placebo-controlled study. *Clin. Invest. Med.* **35,** E1-11 (2012).
29. Lesser, G. J. *et al.* A randomized, double-blind, placebo-controlled study of oral coenzyme Q10 to relieve self-reported treatment-related fatigue in newly diagnosed patients with breast cancer. *J. Support. Oncol.* **11,** 31–42 (2013).
30. Littlewood, T. J., Kallich, J. D., San Miguel, J., Hendricks, L. & Hedenus, M. Efficacy of darbepoetin alfa in alleviating fatigue and the effect of fatigue on quality of life in anemic patients with lymphoproliferative malignancies. *J. Pain Symptom Manage.* **31,** 317–325 (2006).
31. Mar Fan, H. G. *et al.* A randomised, placebo-controlled, double-blind trial of the effects of d-methylphenidate on fatigue and cognitive dysfunction in women undergoing adjuvant chemotherapy for breast cancer. *Support. Care Cancer* **16,** 577–583 (2008).
32. Mitchell, G. K. *et al.* The effect of methylphenidate on fatigue in advanced cancer: An aggregated N-of-1 trial. *J. Pain Symptom Manage.* **50,** 289–296 (2015).
33. Norager, C. B., Jensen, M. B., Madsen, M. R., Qvist, N. & Laurberg, S. Effect of darbepoetin alfa on physical function in patients undergoing surgery for colorectal cancer. A randomized, double-blind, placebo-controlled study. *Oncology* **71,** 212–220 (2006).
34. Page, B. R. *et al.* Phase II double-blind placebo-controlled randomized study of armodafinil for brain radiation-induced fatigue. *Neuro. Oncol.* **17,** 1393–1401 (2015).
35. Palesh, O. G. *et al.* Impact of paroxetine on sleep problems in 426 cancer patients receiving chemotherapy: a trial from the University of Rochester Cancer Center Community Clinical Oncology Program. *Sleep Med.* **13,** 1184–1190 (2012).
36. Morrow, G. R. *et al.* Differential effects of paroxetine on fatigue and depression: a randomized, double-blind trial from the University of Rochester Cancer Center Community Clinical Oncology Program. *J. Clin. Oncol.* **21,** 4635–4641 (2003).
37. Paulsen, O. *et al.* Efficacy of methylprednisolone on pain, fatigue, and appetite loss in patients with advanced cancer using opioids: a randomized, placebo-controlled, double-blind

trial. *J. Clin. Oncol.* **32,** (2014).

38. Ribeiro, S. M. de F. *et al.* Effects of zinc supplementation on fatigue and quality of life in patients with colorectal cancer. *Einstein (Sao Paulo).* **15,** 24–28 (2017).
39. Richard, P. O., et al. (2014). 'A phase II, randomized, double-blind, placebocontrolled trial of methylphenidate for reduction of fatigue in prostate cancer patients receiving LHRH-agonist therapy.' *J. Urol. 191(4 SUPPL. 1) e768.*
40. Richard, P. O. *et al.* Phase II, randomised, double-blind, placebo-controlled trial of methylphenidate for reduction of fatigue levels in patients with prostate cancer receiving LHRH-agonist therapy. *BJU Int.* **116,** 744–52 (2015).
41. Roscoe, J. A. *et al.* Effect of paroxetine hydrochloride (Paxil) on fatigue and depression in breast cancer patients receiving chemotherapy. *Breast Cancer Res. Treat.* **89,** 243–249 (2005).
42. Schmidt, S., et al. (2012). 'A randomized double-blind placebo-controlled crossover study of modafinil in the treatment of fatigue in cancer patients.' *Palliat. Med. 26(4) 416.*
43. Tanioka, H. *et al.* Prophylactic Effect of Dexamethasone on Regorafenib-Related Fatigue and/or Malaise: A Randomized, Placebo-Controlled, Double-Blind Clinical Study in Patients with Unresectable Metastatic Colorectal Cancer (KSCC1402/HGCSG1402). *Oncology* **94,** 289–296 (2018).
44. Yennurajalingam, S. *et al.* High-Dose Asian Ginseng (Panax Ginseng) for Cancer-Related Fatigue: A Preliminary Report. *Integr. Cancer Ther.* **14,** (2015).
45. Yennurajalingam, S. *et al.* A Double-Blind, Randomized, Placebo-Controlled Trial of Panax Ginseng for Cancer-Related Fatigue in Patients With Advanced Cancer. *J. Natl. Compr. Canc. Netw.* **15,** 1111–1120 (2017).

Table 2. Descriptive summary of study characteristics

Continuous Variables	N studies	Min.	1st quartile	Median	Mean	3rd quartile	Max.
Participants in placebo arm	23	12	19	50	68.65	70	316
Number of trial centres	17	1	2	2	8.94	18	40
Mean age of participants	15	53.2	56.15	62	61.96	67.4	71
Percentage of male participants	21	0	34.1	43	49.53	60	100
Duration (in weeks) of intervention	23	1	2	4	4.43	6	10

Roji R, *et al. BMJ Support Palliat Care* 2020; 10:385–394. doi: 10.1136/bmjspcare-2019-002163

Table 3 .Details of categorical and binary variables used in meta-regression

	n	%
**Subjects in placebo arm**		
<=50	12	52.2
51-199	9	39.1
>=200	2	8.7
total	23	100
**Type of Intervention**		
psychostimulants	11	47.8
dietary supplements	2	8.7
antidepressants	2	8.7
herbs	4	17.4
Hormones	1	4.3
Others	2	8.7
steroids	1	4.3
total	23	100
**Type of Study**		
2 arms, no X-over	18	78.3
3 arms	0	0
4 arms	1	4.3
X-over, endpoint before x-over	2	8.7
X-over with washout period	2	8.7
total	23	100
**Tool**		
FACIT-F	11	47.8
BFI	6	26.1
MFSI-SF	1	4.3
CSI	1	4.3
FACT-An	1	4.3
item 3 of BFI	1	4.3
MFI-20	1	4.3
MAF	1	4.3
total	23	100
**Tool Item**		
one item	2	9.1
multidimensional	20	90.9
total	22	100
**Placebo Dosage**		
Less than once a day	1	4.5
Once a day	6	27.3
Twice a day	10	45.5
3 a day	0	0
progressive dosage	4	18.2
prn	1	4.5
total	22	100
**Placebo administration via**		
Oral	21	95.5
intravenous	0	0
IM	1	4.5
total	22	100
**Stage**		
curative	9	47.4
palliative	10	52.6
survivors	0	0
total	19	100
**Placebo dosage regimen**		
fixed-dose	15	68.2
flexible-dose	7	31.8
total	22	100
**Drug placebo significant difference?**		
yes	4	22.2
no	14	77.8
total	18	100
**Risk of Bias RoB2**		
low	9	39.1
unclear	8	34.8
high	6	26.1
total	23	100
